# The Medical Congress

**Published:** 1887-10

**Authors:** 


### THE MEDICAL CONGRESS.

Our senior editor, in the above report of his trip to the International Congress, has given his feelings, excited by the presence of medical gentlemen from the North and from foreign nations, and as drawn out by the interchange of opinions at the various places which he visited.

The subject of the war, the relations which existed between the members of the profession and the subsequent dissensions as connected with the present organization of the Medical Congress, all came up for review. A general regret was manifested that there should ever have been any trouble with us in the outset, but much satisfaction expressed at its happy termination, as evinced by the great success of the Congress.

It must be concluded that if all had been actuated with the patriotic views so well expressed by our senior no dissensions would ever have occurred and no heartburnings or scars of jealously would have ever been left to mar the happiness of any member of the profession.

For want of space we have omitted, until next issue, the part of the report relating to the special work of the Congress—what others said and done.

